# Fluorophotometric Assessment of Tear Volume and Turnover Rate in Healthy Dogs and Cats

**DOI:** 10.1089/jop.2019.0038

**Published:** 2019-11-04

**Authors:** Lionel Sebbag, Rachel A. Allbaugh, Rita F. Wehrman, Lisa K. Uhl, Gil Ben-Shlomo, Thomas Chen, Jonathan P. Mochel

**Affiliations:** ^1^ Department of Veterinary Clinical Sciences, Iowa State University, College of Veterinary Medicine, Ames, Iowa.; ^2^ Department of Biomedical Sciences, SMART Pharmacology, Iowa State University, College of Veterinary Medicine, Ames, Iowa.; ^3^ Department of Small Animal Clinical Sciences, The University of Tennessee, College of Veterinary Medicine, Knoxville, Tennessee.

**Keywords:** tear film, drainage, canine, feline, tear flow, fluorophotometry

## Abstract

***Purpose:*** The study establishes normative data of tear volume (TV) and tear turnover rate (TTR) in healthy dogs and cats, 2 species commonly used for translational research in ophthalmology.

***Methods:*** Thirty-six dogs and 24 cats were enrolled, encompassing a variety of breeds with diverse skull conformations (brachycephalic, mesocephalic, and dolichocephalic). Two microliters of 10% fluorescein were instilled onto the upper bulbar conjunctiva of both eyes, followed by tear collection with 2-μL capillary tubes at 0, 2, 4, 6, 10, 15, and 20 min. Fluorescein concentrations were measured with a computerized scanning ocular fluorophotometer. The TV and TTR were estimated based upon nonlinear mixed-effects analysis of fluorescein decay curves.

***Results:*** In dogs, median (interquartile range) TV, basal TTR (bTTR), and reflex TTR (rTTR) were 65.3 μL (42.3–87.9), 12.2%/min (3.7–22.1), and 50.0%/min (25.9–172.3), respectively. In cats, median (interquartile range) TV, bTTR, and rTTR were 32.1 μL (29.5–39.9), 10.9%/min (3.0–23.7), and 50.0%/min (28.4–89.4), respectively. Body weight (*r* = 0.44) and age (*r* = 0.30) were positively correlated (*P* ≤ 0.019) with TV in dogs. Age was negatively correlated (*P* ≤ 0.018) with TTR in dogs (*r* = −0.33) and cats (*r* = −0.24). However, TV and TTR were not associated with skull conformation in either species.

***Conclusions:*** Dogs have greater TV than cats but similar basal and rTTR. Tear parameters were impacted by body weight and age, but not by skull conformation. In both clinical and research settings, successive lacrimal tests should be spaced by ≥10 min to provide sufficient time for the tear film to replenish, as bTTR is ∼11%/min–12%/min in both species.

## Introduction

Tear fluid dynamics, or the balance between tear secretion, distribution, absorption, evaporation, and drainage, are critical for the maintenance of ocular surface health.^1^ Tear volume (TV) and tear turnover rate (TTR) are parameters that provide insight into these complex dynamics, and as such are valuable for numerous clinical and research applications: for instance (1) TTR helps differentiate between aqueous-deficient dry eye and evaporative dry eye in human patients^2^; (2) TTR impacts the quantity of various tear components such as electrolytes and proteins^3–5^; and (3) determination of TV in horses highlights a large dilution effect of tear fluid on exogenously applied drugs, whereby over half of the drug concentration is diluted immediately upon topical instillation onto equine eyes.^6^

Dogs and cats, in addition to being the most common companion animals worldwide, are commonly used as animal models for translational ocular surface research, as exemplified by canine keratoconjunctivitis sicca^7^ and feline epithelial wound healing.^7,8^ However, information about tear fluid dynamics is lacking in these species, despite numerous reports in humans^1,2,9^ and various animals such as rabbits,^10^ horses,^6^ and cows.^11^ Evaluation of tear dynamics in dogs and cats is likely confounded by their diversity in facial conformations. Indeed, brachycephaly (foreshortening of the facial skeleton) can impact tear drainage in dogs^12^ and in cats,^13^ and the associated lagophthalmos of some brachycephalic animals can impact tear distribution and evaporation.

This study establishes normative data of TV and TTR in dogs and cats using fluorophotometry, a method considered to be the gold standard in assessing tear dynamics.^6,9^ A secondary objective is to determine the impact of cephalic conformation and other variables (age, body weight, and Schirmer values) on canine and feline tear dynamics.

## Methods

### Animals

Thirty-six dogs (*n* = 72 eyes) and 24 cats (*n* = 48 eyes) were enrolled in the study. Before study participation, a consent form was signed by owners, and each subject was confirmed to be ophthalmoscopically healthy by slit-lamp examination, indirect ophthalmoscopy, rebound tonometry (TonoVet; Icare Finland Oy, Espoo, Finland), and normal Schirmer test values (≥15 mm/min in dogs, ≥9 mm/min in cats).^7,14^

A soft measuring tape was used to measure the skull width (widest interzygomatic distance), skull length (dorsal tip of the nose to occipital protuberance), and muzzle length (dorsal tip of the nose to the stop). These values were used to calculate the cephalic index (CI = skull width/skull length) and the craniofacial ratio (CFR = muzzle length/skull length) in each animal.^15–17^ The CI was used to characterize canine subjects as brachycephalic (*n* = 10), mesocephalic (*n* = 16), or dolichocephalic (*n* = 10), as described by Evans and De Lahunta.^15^ Since similar numerical features are lacking in cats, the CI was still utilized for data analysis, but feline subjects were classified as brachycephalic (*n* = 9; eg, Persian, Himalayan, Exotic Shorthair) or nonbrachycephalic (*n* = 15; eg, Domestic Shorthair, Bengal) based on previous studies.^13,17^

The study was approved by the Institutional Animal Care and Use Committee of Iowa State University, and was conducted in accordance with the Association for Research in Vision and Ophthalmology statement for the use of animals in ophthalmic and vision research.

### Fluorophotometry

Fluorophotometry was performed as previously described with minor modifications.^6^ In brief, 2 μL of 10% sodium fluorescein (Akorn, Inc., Buffalo Grove, IL) was instilled onto the dorsal bulbar conjunctiva of each eye using a pipette, with care not to touch the ocular surface. Immediately after 3 manual eyelid blinks (0 min), a 2-μL capillary glass tube (Drummond Scientific Co., Broomhall, PA) was placed in contact with the inferior tear lake for ≤2 s to collect a tear sample. Eyes were then allowed to blink naturally, and tear samples were collected in a similar manner at 2, 4, 6, 10, 15, and 20 min.

A maximum of 2 s was selected for tear collection as a compromise to obtain sufficient tear fluid for analysis while minimizing the risk of inadvertently touching the ocular surface and causing reflex tearing. This duration, however, was often insufficient to completely fill the 2-μL capillary tubes with tears. Thus, after each collection, the length of fluid contained within the capillary tube was measured to the nearest millimeter using a ruler, a value extrapolated to the volume of tears collected, given that the 2-μL tube is 32 mm in length. The content of each capillary tube was expelled into a 2-mL Eppendorf tube prefilled with 1 mL of phosphate-buffered saline (Gibco^®^ PBS, pH 7.2; Thermo Fisher Scientific, Rockford, IL). An additional 1 mL of PBS was added to each tube, followed by vortex mixing for 30 s and transfer to a glass cuvette.

Fluorescein concentration was measured in each sample with a computerized scanning ocular fluorophotometer (Fluorotron Master™; Coherent Radiation, Palo Alto, CA). All tear sample collection and experiments were performed in the morning (from 8 am to 12 pm) to reduce the potential impact of circadian rhythm on TTR.^18^ All subjects were confirmed to be free of corneal epithelial defects using a cobalt blue light evaluation at completion of the last tear collection. Of note, both eyes of each animal received the same amount of fluorescein at baseline, except for 6 beagle dogs in whom one random eye received 2 μL of 10% fluorescein^6^ while the other eye received 1 μL of 1% fluorescein^19^—an experiment conducted to assess the effect of fluorescein dosing on tear dynamics.

### Data analysis

Since the fluorophotometer output is nonlinear at high fluorescein concentrations, a calibration curve was established for the Fluorotron Master by analyzing a dilution series of known fluorescein concentrations in triplicate (1–10,000 ng/mL).^20,21^ Fluorescein concentrations in tear samples were corrected based on the resulting calibration equation (*y* = −4E−05*x*^2^ + 0.9567*x* + 20.581).

Fluorescein data of each animal were inputted to Monolix^®^ version 2018R2 (Lixoft, Orsay, France). Selected data points were censored in Monolix when a peak of fluorescence could not be identified on the fluorophotometer reading, or if the tear fluorescein concentration did not make physiologic sense (eg, higher fluorescein at 2 min compared with baseline).^6^ Overall, <10% (93/944) of all data points were left censored.

Mathematical models of fluorescein disposition time course were written as nonlinear mixed-effects (NLME) models as previously described and detailed in Supplementary Appendix SA.^22–25^ Data collection from the left and the right eye practically constitutes a repeat sample from the same individual. To account for this repeat sampling procedure, biological data collected from the right and left eye of each study subject were modeled using a *within-dog* variability term in the statistical model structure. Inclusion of covariate relationships with the model parameters TV and TTR (age, body weight, sex, skull type, CI, CFR, Schirmer tear test value, and amount of fluorescein instilled onto the ocular surface) was assessed for statistical significance using a Pearson correlation test (for continuous variables) or Fisher's exact test (for categorical variables) at a *P* < 0.05 threshold.

**FIG. 1.**
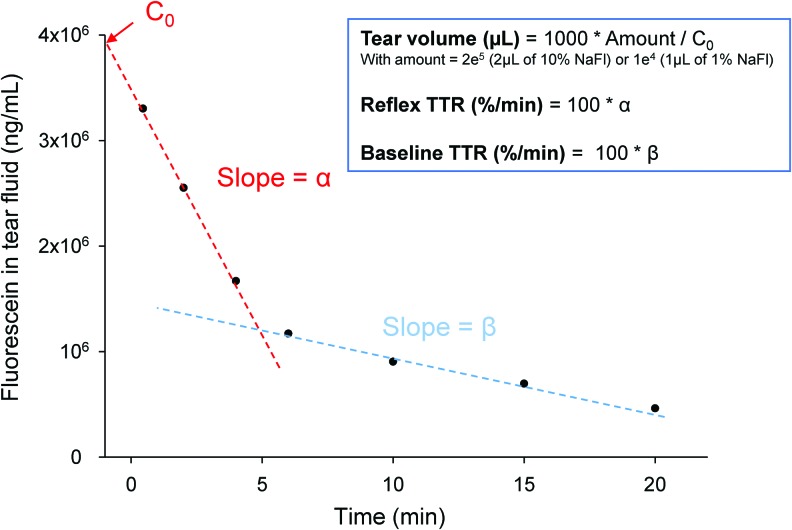
Representative fluorescein decay curve in tear fluid of a dog or a cat, allowing for calculation of tear volume and TTR (reflex and basal) using parameters calculated with nonlinear mixed-effects model. *C*_0_ represents the fluorescein concentration in tear fluid at *t* = 0 min, extrapolated from the fluorescein decay curve. NaFl, sodium fluorescein; TTR, tear turnover rate. Color images are available online.

**FIG. 2.**
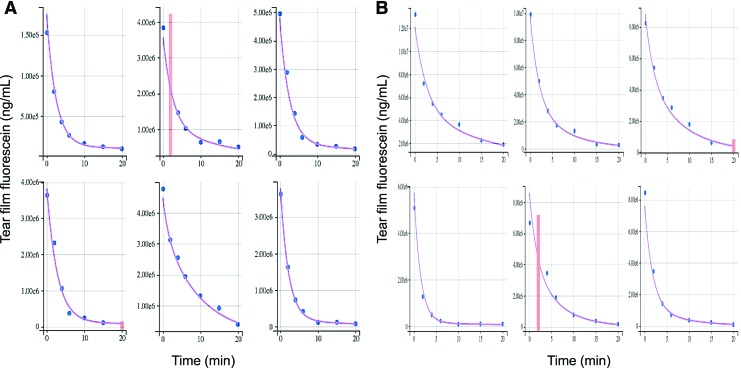
Comparison of predicted tear fluorescence over time (*purple curve*) with observed data (*blue points*) for a random sample of dogs **(A)** and cats **(B)**. Censored data are shown as vertical *red bars*. Color images are available online.

Data modeling best fitted a biphasic decay curve, as previously reported,^1,19,26,27^ allowing for the calculation of the following parameters (Fig. 1): (1) TV, calculated from the monophasic decay of fluorescence in the first regression line after instillation of fluorescein^1,26^; (2) reflex TTR (rTTR), calculated from the slope of the first regression line^27^; and (3) basal TTR (bTTR), calculated from the slope of the second regression line.^1,19,26,27^ bTTR represents tear drainage during nonstimulated physiologic conditions, while rTTR represents the faster drainage that occurs as a response to noxious stimulation or irritative conditions (eg, foreign body, corneal abrasion, and eye drop administration). Data supporting the validity and robustness of the model are shown in Fig. 2 and Supplementary Appendix SA. Finally, theoretical TV was calculated based on tear film thickness measured in a subset of dogs (Supplementary Appendix SB).^6,28,29^

## Results

**FIG. 3.**
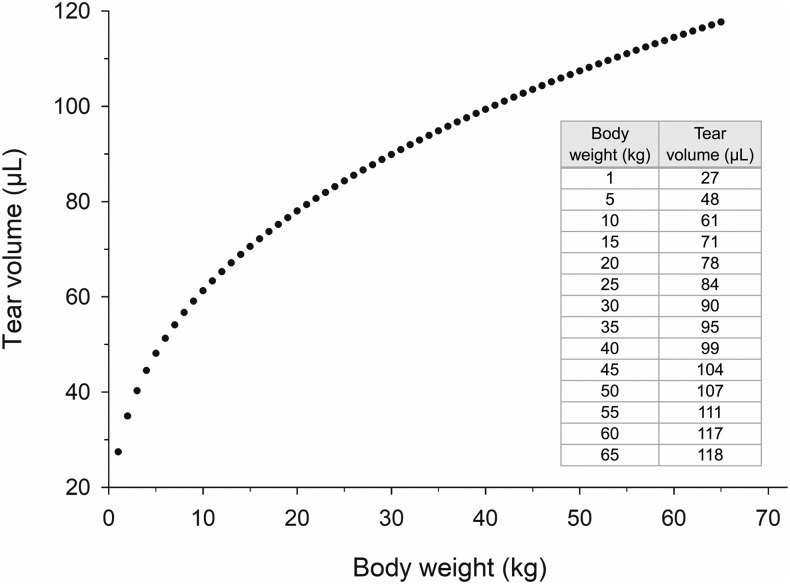
A positive association was found between canine body weight and tear volume (Pearson's correlation test). Estimated tear volumes are described in the table for body weights ranging from 1 to 65 kg.

All eyes (*n* = 72 in dogs, *n* = 48 in cats) were deemed healthy on ophthalmic examination and were utilized for data analysis. Using the model, TV and TTR for the general canine and feline population were calculated, and results are presented as median and interquartile range (25th–75th percentile) in Table 1. Several correlations were found between individual characteristics and tear film parameters (Table 2). In particular, body weight (*r* = 0.44, *P* < 0.001; Fig. 3) and age (*r* = 0.30, *P* = 0.019) were positively correlated with TV in dogs, and a negative correlation was found between age and TTR in dogs (*r* = −0.33, *P* = 0.007) and cats (*r* = −0.24, *P* = 0.018). Further, a positive correlation was detected between the dose (amount of fluorescein instilled onto the ocular surface) and the calculated TV in dogs (*P* < 0.001).

**Table 1.** Normative Data of Tear Volume and Tear Turnover Rate in Dogs and Cats, Presented as Median (In Bold) and Interquartile Range (25th–75th Percentile)

	*Dog (*n* = 72 eyes)*	*RSE (%)*	*Cat (*n* = 48 eyes)*	*RSE (%)*	*Human^1,9,27^ (*n* = 31–74 eyes)*
TV (μL)	**65.3** (42.3–87.9)	7.5	**32.1** (29.5–39.9)	20.3	7.0–12.4
Basal TTR (%/min)	**12.2** (3.7–22.1)	5.4	**10.9** (3.0–23.7)	13.7	10–20
Reflex TTR (%/min)	**50.0** (25.9–172.3)	5.4	**50.0** (28.4–89.4)	13.7	31.5–100

The RSEs of parameter estimates from the model are listed for each species. For comparison, the human tear film parameters are presented in the right column. Values in bold represent the median values for TV, bTTR and TTR.

RSE, relative standard errors; TTR, tear turnover rate; TV, tear volume.

**Table 2.** Correlations Between Tear Parameters and Covariates, Using the Pearson Test for Continuous Variables (Age, Body Weight, STT, CI, CFR) and Fisher's Exact Test for Categorical Variables (Gender, Dose, Skull Type)

	*Age*	*Body weight*	*Gender*	*STT*	*Dose*	*Skull type*	*CI*	*CFR*
*Dog*								
TV	*r* = 0.30 (*P* = 0.019)	*r* = 0.44 (*P* < 0.001)	—	—	*P* < 0.001	—	—	—
TTR	*r* = −0.33 (*P* = 0.007)	—	*P* = 0.005	—	—	—	—	—
*Cat*								
TV	—	—	—	*r* = 0.72 (*P* < 0.001)	N/A	—	—	—
TTR	*r* = −0.24 (*P* = 0.018)	—	—	—	N/A	—	—	—

Pearson's correlation coefficients (*r*) are noted for continuous variables.

—, no correlation found; CFR, craniofacial ratio; CI, cephalic index; N/A, not assessed; STT, Schirmer tear test.

Of note, a *post hoc* sample size calculation (SigmaPlot version 14.0; Systat Software, Point Richmond, CA) showed that *n* = 360 dogs and *n* = 130 cats would be required to detect statistical differences in TV and TTR among animals with diverse skull conformations, assuming a power of 80% and an alpha of 0.05.

## Discussion

This study establishes the tear film dynamics in healthy dogs and cats, accounting for the diversity of skull conformation occurring in these companion animals. Dogs and cats have a few advantages over current preclinical models of ocular surface disease: their ocular anatomy better resembles humans than rabbits or laboratory rodents,^30^ and both species develop spontaneous diseases that share strong similarities with human pathologies (eg, dry eye disease, herpes keratitis).^7,31^

In pharmacology, for instance, extrapolation of findings from rabbits to humans is compromised by significant differences in precorneal residence time of drugs between these 2 species.^32^ In fact, the tear flow in rabbits is much slower than that in humans,^1,10^ a difference likely explained by variability in tear film stability, mucin composition, and blink rate.^20,33,34^ Such differences would be minimized when working with companion animals, since it takes approximately the same amount of time for the tear fluid to replenish in humans as in dogs and cats (5–10 min); indeed, bTTR in dogs (12.2%/min) and cats (10.9%/min) better mimics the human's ocular surface physiology (10%/min–20%/min).^1^

On the contrary, the TV is different among these species: TV in dogs (65.3 μL) and cats (32.1 μL) is larger than that in humans (7.0–12.4 μL),^9,27^ a finding that could explain why Schirmer testing is recommended for 1 min in companion animals versus 5 min in people. Compared with humans, dogs and cats have a larger corneal surface to lubricate (average corneal diameter in humans is 11.7 mm vs. 16.7 mm and 16.5 mm for dogs and cats, respectively),^35–37^ and they possess an additional secretory tissue (gland of the third eyelid) to supplement the main lacrimal gland with aqueous tear production. Differences in tear film thickness [3.4 μm in humans^29^ vs. 15.1 μm in dogs (Supplementary Appendix SB)] could also explain the larger canine TV.

Such differences in lacrimal volume have important practical implications. When compared with humans, dogs and cats have a greater initial dilution of a drug administered onto their ocular surface. Conversely, they offer easier collection of sufficient tear fluid for analytical purposes; in fact, up to 106 and 43 μL can be easily collected within 1 min using ophthalmic sponges in dogs and cats, respectively.^38^

Fluorophotometry is often considered superior to other tear assessment methods, such as fluorescein clearance test or lacrimal scintigraphy,^1,39^ to study tear film dynamics. However, one must be cognizant of the initial amount of fluorescein used in fluorophotometry studies as it can impact the calculated tear dynamics parameters,^18,27^ a finding verified in this study.

Here, we combined this analytical method with detailed modeling of the data to improve the robustness of our findings. Unlike previous studies in which *t* = 5 min is empirically selected as the transition between reflex and basal tearing,^1,9,19,26^ mathematical modeling appreciates the nuances of fluorescein decay between eyes and subjects, while incorporating individual characteristics (such as age and body weight) into the final analysis. Another value of the NLME approach is the ability to model both eyes simultaneously, thereby taking into account within-subjects (between eyes) variability (WSV) in the tear fluid dynamics. This is particularly relevant as this variability was estimated to be fairly high, such that modeling of the data without factoring in WSV could lead to significant model misfits.

Further, we purposely enrolled animals with a variety of cephalic conformations to be representative of the diverse breeds examined by veterinarians and researchers. Brachycephalic animals could theoretically have reduced TTR due to functional punctal occlusion caused by medial entropion and/or altered anatomy of the nasolacrimal duct associated with the skull conformation.^13^ The lack of statistical impact of cephalic conformation on TTR is likely due to strict inclusion criteria (normal subjects without obvious pathological changes) and an overall relatively low sample size. Recruiting brachycephalic animals with healthy ocular surface was indeed rather challenging, particularly in cats.

A few significant correlations were detected between individual characteristics and tear dynamics parameters. Notably, lacrimal volume gets larger with increasing body weight in dogs (Fig. 3), a finding previously documented in juvenile,^40^ but not adult dogs.^41^ Moreover, TTR decreases with age in both dogs and cats. In humans, aging reduces eyelid kinematics (blinking amplitude and peak velocity),^42^ a physiologic change that could result in a less-efficient pump mechanism to drain the tear fluid through the nasolacrimal duct; the same may be true in companion animals.

In both clinical and research settings, we recommend waiting 10 min between successive lacrimal tests. Indeed, 10 min would be required to fully replenish the canine or feline tear film if the initial lacrimal test did not cause ocular irritation (eg, strip meniscometry), as the bTTR is ∼11%/min–12%/min in both species. However, 5 min may be sufficient if the diagnostic test is causing reflex tearing (eg, Schirmer test) given that the initial TTR is very fast (rTTR = 50%/min).

From a pharmacologic standpoint, the concentration of drug instilled onto the ocular surface is immediately diluted by 3-fold in dogs and 2-fold in cats upon mixing with the tear film, assuming an average drop size of 35 μL.^39^ The precorneal residence time of this drug is expected to be <10 min as TTR after eye drop administration is presumably faster than that under physiologic conditions.^32^ Further, fluorophotometry data from healthy animals can be compared with clinical cases with ocular surface disease, helping to differentiate between symptomatic and asymptomatic patients (TTR is significantly lower in symptomatic patients)^43^—particularly in cats for whom clinical signs of aqueous tear deficiency are not as overt as in dogs^44^—and between aqueous-deficient and evaporative dry eye (TTR is significantly lower in aqueous deficiency),^45^ a distinction well established in human patients but poorly characterized in veterinary medicine.

The main limitation of our study is the relatively low sample size, which could explain the lack of significant effect of the skull type (brachycephalic, mesocephalic, and dolichocephalic) on tear film dynamics. Further, *in situ* assessment of tear fluorescence—as described in most investigations on human subjects^9,19,21,27^—was not possible in our study, as dogs and cats would not tolerate the 20-min protocol without heavy sedation or general anesthesia, which would in turn affect tear film dynamics. Thus, tear fluid had to be collected with capillary tubes before analysis, and this could have added another source of variability in the fluorophotometry measurements.

The volume of fluid collected was calculated in each sample, but the exact duration of tear collection was not standardized among subjects. Since collection duration did not exceed 2 s to avoid reflex tearing, the potential impact of sampling duration on fluorophotometry data is deemed negligible in this study; however, this parameter should be recorded in future studies should longer collection duration be necessary (ie, higher risk of inadvertent reflex tearing).

In conclusion, the normative data established in this study have several implications for both clinicians and researchers. In particular, successive lacrimal tests should be spaced by ≥10 min to provide sufficient time for the tear film to replenish, as bTTR is ∼11%/min–12%/min in both species. In both species, tear film parameters were affected by body weight and age, but not by skull conformation.

## Supplementary Material

Supplemental data

## Supplementary Material

Supplementary Appendix SA

Supplementary Appendix SB
