# Supplementary material for: Fluorophotometric Assessment of Tear Volume and Turnover Rate in Healthy Dogs and Cats
Source: J Ocul Pharmacol Ther. 2019 Nov 4;35(9):497–502. doi: 10.1089/jop.2019.0038 (PMC6842130; doi:10.1089/jop.2019.0038)
Supplement: Supplemental data [file Supp_Data.pdf]

Supplementary Data

Supplementary Appendix A. Mathematical Modeling Using Nonlinear Mixed Effects

Parameters estimation was performed using the stochastic approximation expectation maximization (SAEM) algorithm for nonlinear mixed-effects models as implemented in the Monolix Suite. Competing models were evaluated numerically using Bayesian information criteria and precision of parameter estimates—defined as relative standard error (RSE) of the estimate. Standard goodness-of-fit diagnostics, including observed versus predicted fluorescein concentrations, individual fits, and weighted residuals time course were used to graphically assist comparison, as previously described.[S1–S4] SAEM convergence and final model parameterization were assessed graphically by inspection of search stability, distribution of the individual parameters, distribution of the random effects, individual prediction versus observation, individual fits, and distributions of the weighted residuals. The numerical precision of parameter estimates was assessed using RSE. The numerical normality of individual parameters was assessed using a Shapiro–Wilk test for normality. The normality of the distribution of residuals was assessed using a Shapiro–Wilk test, and the centering of the distribution of residuals (ie, 0) was assessed using a Van Der Waerden test. P values <0.05 were considered as statistically significant.

A suitable mathematical model has the following features (Supplementary Appendix Figures SA1–SA4): (1) the line of identity is aligned with the regression line, while (2) the residues (differences between observations and predictions) are centered on a mean value of 0, with (3) a homogeneous dispersion around the mean.

Supplementary Appendix B. Tear Film Thickness and Theoretical Tear Volume

Tear thickness was measured with spectral-domain optical coherence tomography (SD-OCT; Optovue iVue, Fremont, CA) in 6 beagle dogs, selected based on their homogeneous subject characteristics (eg, similar breed, age, skull type, body weight, etc.). Dogs were manually restrained with their eyelids held open by an assistant. Tear thickness was measured with ImageJ software (National Institutes of Health, Bethesda, MD) from images captured with the SD-OCT. The average tear thickness from all eyes ($d = 0.01512\,\text{mm}$) was used to calculate the theoretical canine tear volume (TV) based on a mathematical equation described in a previous publication,[S5] assuming a radius of the canine globe (r) of 10.45 mm.[S6]

$$\text{TV}\ (\mu\text{L}) = \frac{1}{2}\left\{\frac{4}{3}\pi(r+d)^3\right\} - \frac{1}{2}\left\{\frac{4}{3}\pi r^3\right\}.$$

Similar to horses,[S5] the theoretical TV value calculated with this method (10.4 μL) was lower than the one calculated with fluorophotometry (59 μL for a median beagle body weight of 9 kg), a finding likely explained by the unevenness of tear film thickness on the ocular surface. The mathematical equation assumes the tear film thickness to be homogeneous when in reality tears are pooling in conjunctival fornices and tear film is much thicker at the lower and upper tear menisci.[S7] However, the tear menisci could not be readily imaged in our dogs as we did not use sedation or general anesthesia.

Supplementary References

S1. Riviere, J.E., Gabrielsson, J., Fink, M., and Mochel, J.P. Mathematical modeling and simulation in animal health. Part I: moving beyond pharmacokinetics. *J. Vet. Pharmacol. Ther.* 39:213–223, 2016.

S2. Mochel, J.P., and Danhof, M. Chronobiology and pharmacologic modulation of the renin-angiotensin-aldosterone system in dogs: What have we learned? *Rev. Physiol. Biochem. Pharmacol.* 169:43–69, 2015.

S3. Mochel, J.P., Fink, M., Peyrou, M., Soubret, A., Giraudel, J.M., and Danhof, M. Pharmacokinetic/Pharmacodynamic modeling of renin-angiotensin aldosterone biomarkers following angiotensin-converting enzyme (ACE) inhibition therapy with benazepril in dogs. *Pharm. Res.* 32:1931–1946, 2015.

S4. Fink, M., Letellier, I., Peyrou, M., et al. Population pharmacokinetic analysis of blood concentrations of robenacoxib in dogs with osteoarthritis. *Res. Vet. Sci.* 95:580–587, 2013.

S5. Chen, T., and Ward, D.A. Tear volume, turnover rate, and flow rate in ophthalmologically normal horses. *Am. J. Vet. Res.* 71:671–676, 2010.

S6. Salgüero, R., Johnson, V., Williams, D., et al. CT dimensions, volumes and densities of normal canine eyes. *Vet. Rec.* 176:386, 2015.

S7. Wang, J., Aquavella, J., Palakuru, J., Chung, S., and Feng, C. Relationships between central tear film thickness and tear menisci of the upper and lower eyelids. *Invest. Ophthalmol. Vis. Sci.* 47:4349–4355, 2006.

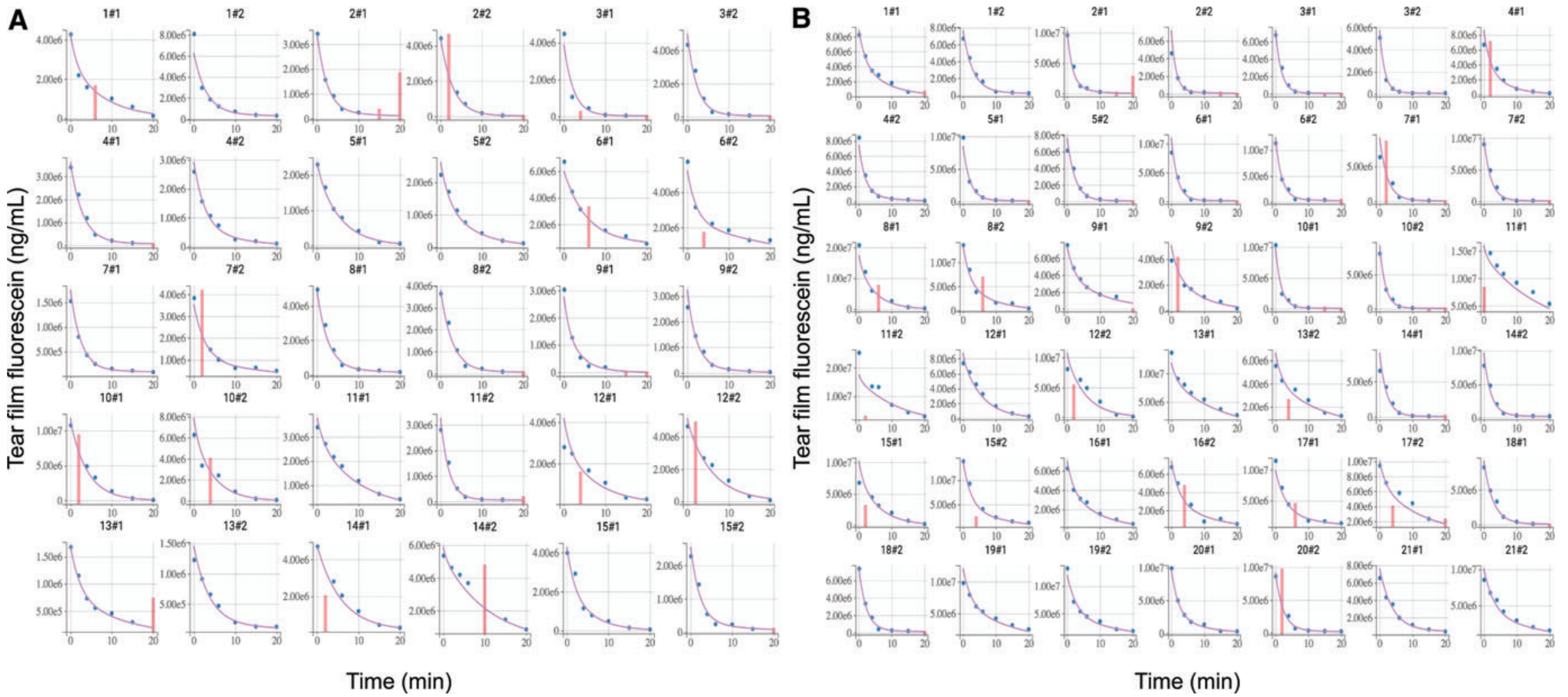

SUPPLEMENTARY APPENDIX FIG. A1. Comparison of predicted tear fluorescence over time (*purple curve*) with observed data (*blue* points) for a random sample of dogs **(A)** and cats **(B)**. Censored data are shown as vertical *red bars*. The ID of each individual is listed above the individual fit, followed by #1 for the right eye and #2 for the left eye.

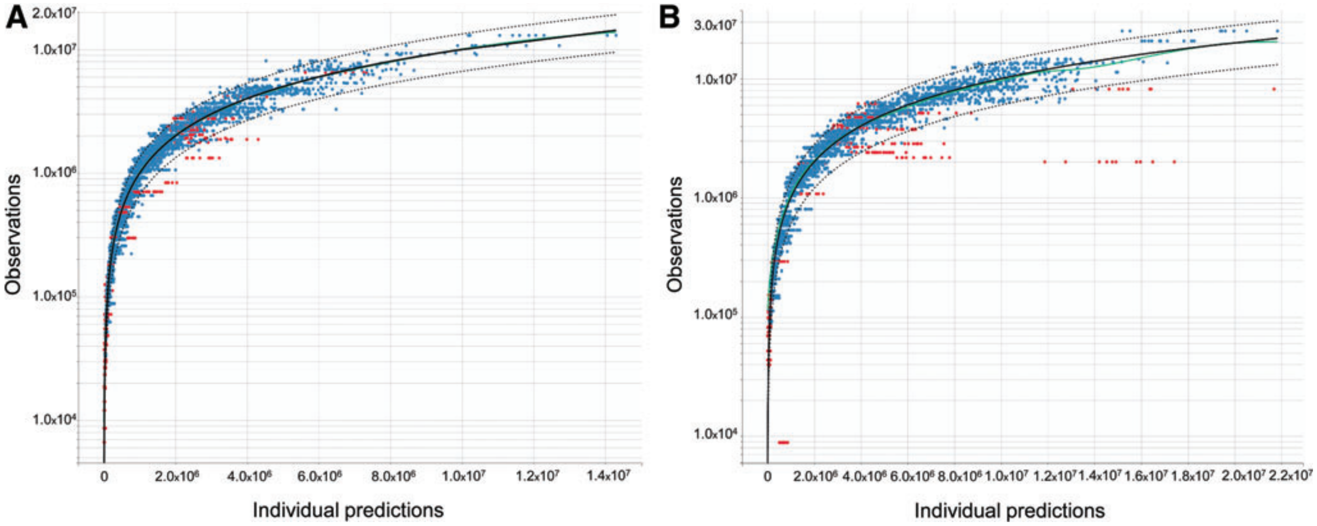

SUPPLEMENTARY APPENDIX FIG. A2. Standard goodness-of-fit diagnostics: individual predictions versus observations (log10) for the fluorophotometry data in dogs **(A)** and cats **(B)**. Observed data are depicted with *blue dots*; the *solid black line* represents the identity line; the regression line is portrayed in light *green color*; censored data points (<10%) are represented with *red dots*.

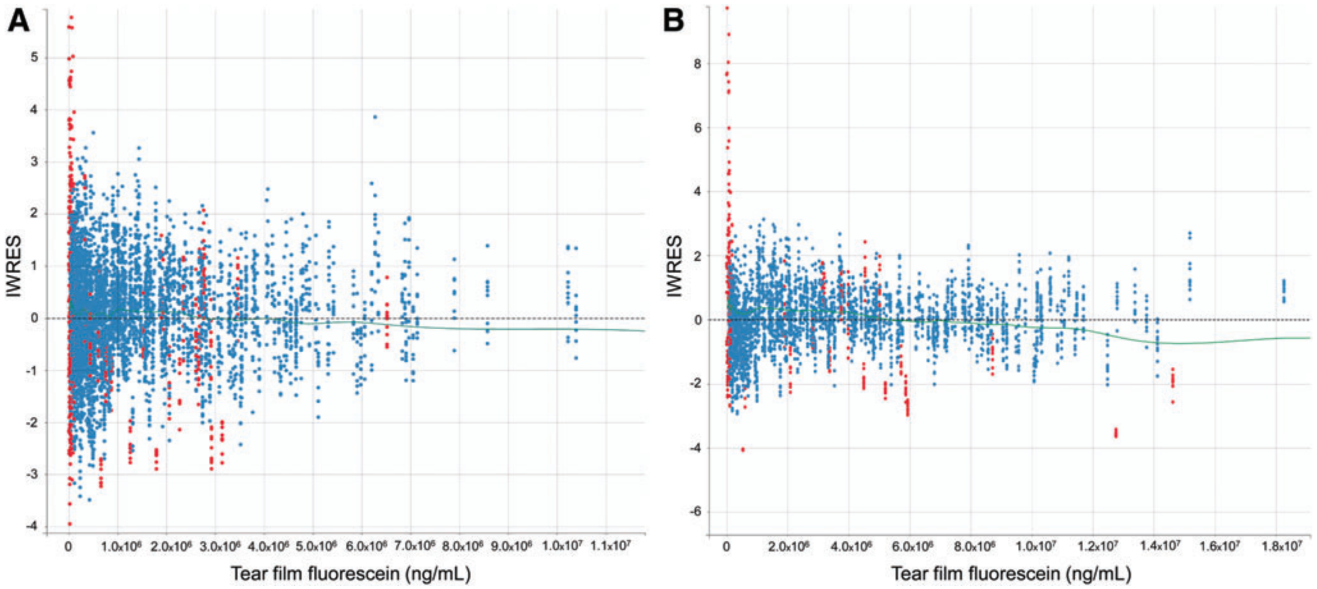

SUPPLEMENTARY APPENDIX FIG. A3. IWRES versus fluorescein concentrations in tears of dogs **(A)** and cats **(B)**. Observed data are depicted with *blue dots*. Censored data points (<10%) are represented with *red dots*. The turquoise line represents the spline (loess regression). IWRES, individual weighted residuals.

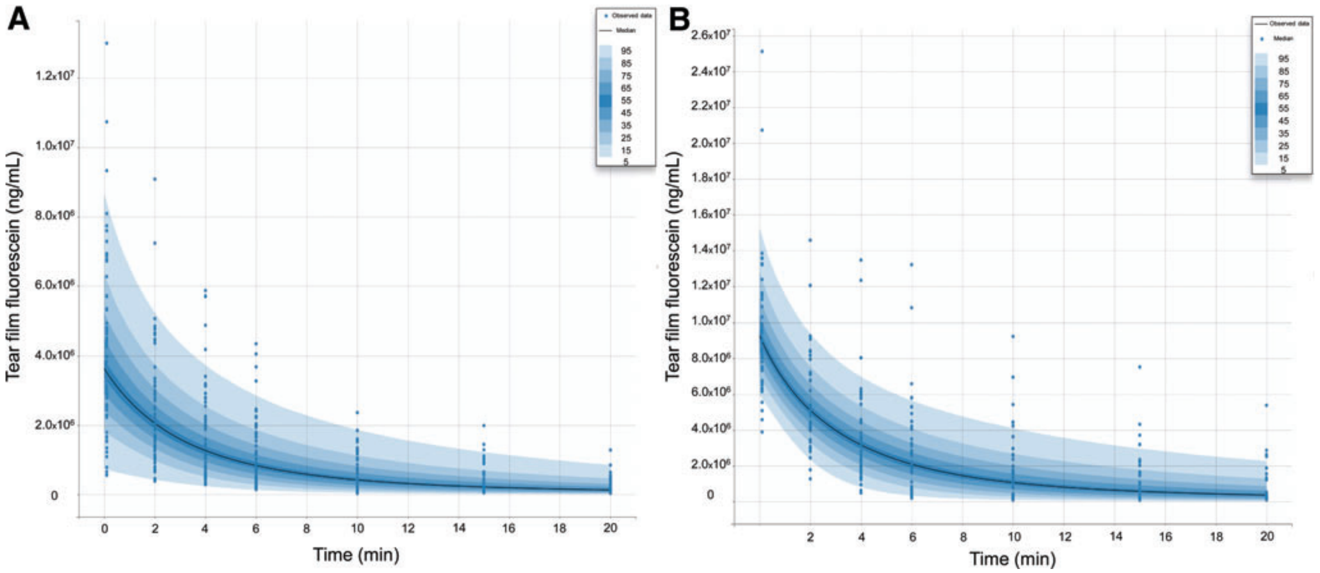

SUPPLEMENTARY APPENDIX FIG. A4. Simulations of fluorescein vs. time disposition from 500 Monte Carlo simulations using final parameter estimates from the NLME model. Predictions derived from the 5th to the 95th percentile of the model simulations were able to reproduce the variability in the observed data from the original population of dogs (A) and cats (B). NLME, nonlinear mixed effects.
